# Determinants of multidrug-resistant tuberculosis in patients who underwent first-line treatment in Addis Ababa: a case control study

**DOI:** 10.1186/1471-2458-13-782

**Published:** 2013-08-28

**Authors:** Selamawit Hirpa, Girmay Medhin, Belaineh Girma, Muluken Melese, Alemayehu Mekonen, Pedro Suarez, Gobena Ameni

**Affiliations:** 1 College of Health Sciences, Adama Science and Technology University, P. O. Box 396, Adama, Ethiopia; 2 Aklilu Lemma Institute of Pathobiology, Addis Ababa University, P. O. Box 1176, Addis Ababa, Ethiopia; 3 Management Sciences for Health, HEAL-TB Project, P. O. Box 1157, Addis Ababa, Ethiopia; 4 College of Health Sciences, School of Public Health, Addis Ababa University, P. O. Box 1176, Addis Ababa, Ethiopia; 5 Management Sciences for Health, Arlington, VA, USA

**Keywords:** TB, MDR-TB, TB treatment, TB treatment regimens, Adherence to TB treatment, TB treatment failure, DOTS

## Abstract

**Background:**

Worldwide, there were 650,000 multidrug-resistant tuberculosis (MDR-TB) cases in 2010, and in 2008 the World Health Organization estimated that 150,000 deaths occurred annually due to MDR-TB. Ethiopia is 15^th^ among the 27 MDR-TB high-burden countries. This study identifies factors associated with the occurrence of MDR-TB in patients who underwent first-line TB treatment in Addis Ababa City.

**Methods:**

A case control study was conducted at St. Peter Hospital and five health centers in Addis Ababa from 1 November 2011 to February 30, 2012. Cases were MDR-TB patients who were confirmed with culture and drug-susceptibility testing and were in treatment at St. Peter Hospital during the study period. Controls were patients who were on first-line anti-TB treatment and were registered as cured or having completed treatment in the period 9 April 2009– 28 February 2010, in five health centers of Addis Ababa City. Accordingly, 134 cases and an equal number of controls were included in this study. A structured interview questionnaire was used to assess factors that could potentially be associated with the occurrence of MDR-TB.

**Results:**

Factors that were significantly associated with MDR-TB: drug side effects during first-line treatment (adjusted odds ratio (AOR): 4.5, 95% CI; 1.9 - 10.5); treatment not directly observed by a health worker (AOR = 11.7, 95% CI; 4–34.3); interruption of treatment of at least a day (AOR = 13.1, 95% CI 3.0-56.6); duration of treatment between 2 and 7 months (AOR = 14.8, 95% CI 2.3-96.4); and retreatment with the Category II regimen (P = 0.000). In the current study, HIV infection was not significantly associated with the occurrence of MDR-TB.

**Conclusions:**

Patients who were not in strict DOTS programs and did not adhere to first-line TB treatment and patients who experienced side effects during first-line treatment and Category II retreatment were at significantly increased risk of developing MDR-TB. The DOTS program should, therefore, be strengthened to increase patient adherence. Drug-susceptibility testing is also highly recommended for all Category I treatment regimen failures before those patients begin the Category II regimen.

## Background

Multidrug-resistant tuberculosis (MDR-TB) is a type of TB that is resistant to at least the first line anti-TB drugs, Rifampacin and Isoniazid. MDR-TB occurs either when a person is infected with a resistant strain or when improper treatment leads to drug selection of the resistant strain [1]. When an individual who has no history of first-line TB treatment develops MDR-TB, it is termed primary. When insufficient treatment leads to selection of spontaneously resistant strains (i.e., drug resistance is acquired), the disease is termed secondary MDR-TB [2].

Worldwide, there were 650,000 MDR-TB cases in 2010, and in 2008 World Health Organization (WHO) estimated that there were 150,000 deaths annually due to MDR-TB [3]. Overall, the 27 high MDR-TB burden countries accounted for 85% of all MDR-TB cases. China, and India, was the top two countries accounting 50% MDR-TB cases [3]. A 2010 WHO report showed that the number of MDR-TB cases is rising in Africa [1]. Ethiopia is 15^th^ among the 27 MDR-TB high-burden countries, with an estimated 5,200 cases occurring each year [4].

The occurrence of MDR- TB is mainly attributable to human error, although genetic factors are also believed to contribute to a certain extent [5]. The principal patient-related factor that predicts the occurrence of MDR-TB is non-adherence to treatment [6]. The first-line anti-TB drugs used in Ethiopia in 2009/2010 were rifampicin (R), ethambutol (E), isoniazid (H), and pyrazinamide (Z) [7]. The category II treatment regimen (S (ERHZ) for two months, ERHZ for one month and E (RH) for five months three times a week), which adds streptomycin to the category I regimen (ERHZ for two months and RH for four months) has been blamed for increasing the risk of developing MDR-TB [8], despite the fact that patients have already been exposed to most of the drugs.

The emergence of MDR-TB is a threat for the populations of resource-limited countries. In Ethiopia, the low socioeconomic status of the people, high prevalence of infectious diseases and limited access to well-equipped health care facilities worsens the effect of MDR-TB. Furthermore, poor treatment outcomes, longer treatment time (about two years), higher treatment costs, and many more complications make MDR-TB a more complex disease than TB [1,9]. In 2010, less than 5% of new and previously treated TB patients were tested for MDR-TB because of limited availability of the test in most developing countries [10]. For example in Ethiopia, in 2010 the ratio of laboratories capable of performing mycobacterial culture was 0.1 per 5 million populations [10]. Similarly, the ratio of laboratories capable of running line probe assays (LPA) for rapid detection of MDR-TB was 0.1 per 5 million populations [10]. At the time of this study in Ethiopia, the LPA, or culture using Lőwenstein-Jensen media (LJ), and drug-susceptibility testing (DST) were provided only at the Ethiopian Health Nutrition and Research Institute (EHNRI) in Addis Ababa.

MDR-TB occurs mostly in relation to improper treatment of drug-susceptible TB. In countries like Ethiopia MDR-TB is becoming a challenge because of poor adherence to treatment and an increase in the use of illegal and unapproved treatment regimens for MDR-TB [9]. To make things worse, in these TB and MDR-TB high-burden countries patients stay in their communities for longer periods without being diagnosed or getting proper treatment. Even after diagnosis, because there are few diagnostic and treatment facilities and a lack of trained health professionals and drugs, patients do not start treatment immediately. This delay potentially allows easy spread of the disease to a large number of individuals within a short time. The aim of this study is to asses factors that determine the occurrence MDR-TB among patients who had taken first line anti-TB treatment in Addis Ababa City.

## Methods

### Study area and study design

This health institution–based case control study was conducted between 1 November 2011 and 28 February 2012 in Addis Ababa, the capital city of Ethiopia. The estimated population size of Addis Ababa is 2.74 million and the male population constitutes 48% [11]. Administratively, the city is divided into 10 sub-cities and further classified into 99 *kebeles* (lowest government administrative unit). The health institutions in the city includes 47 hospitals, 204 private higher clinics, 226 private mid-level clinics (known as medium clinics), 143 private lower clinics, and 37 government health centers [Addis Ababa Health Bureau report].

### Study setting

Cases were selected from St. Peter Hospital, one of the two MDR-TB patient treatment centers in Addis Ababa, and controls were selected from Addis Ketema Health Center in Addis Ketema sub-city; Woreda 9 Health Centers in Kolfe Keranyo sub-city; Lideta Health Center in Lideta sub-city; Kasanches Health Center in Kirkos sub-city; and Woreda 19 and Nifas Silk Lafto health centers in Nifas Silk Lafto sub-city.

### Eligibility of study participants

MDR-TB patients diagnosed by LPA, or culture using LJ, and DST at the EHNRI and who were being treated at St. Peter Hospital during the study period were considered as cases. In Ethiopia a patient is a suspect for MDR-TB if he/she is a symptomatic close contact of a confirmed MDR-TB patient; a symptomatic individual from a known high-risk group such as health workers; a case of treatment failure; a new TB patient who remains smear positive after 2 months of treatment (for new cases) and after 3 months of retreatment with first-line treatment or retreatment (e.g., return after default, relapse) [12]. The controls were patients who had completed first-line anti-TB treatment and were declared cured or treatment completed using the WHO criteria and adopted by FMOH of treatment outcomes [13] between 9 April 2009 and 28 February 2010. Additionally, the controls were those with no clinical symptoms of TB based on the WHO criteria.

### Recruitment of study participants

During the study period there were 147 eligible confirmed MDR-TB cases at St. Peter Hospital, 134 of who consented to participate in the study. These patients were residents of Addis Ababa who had a history of taking their first course of first-line TB treatment and were on MDR-TB treatment during the period of data collection. Prior to identification of the controls, five health facilities were identified based on the number of MDR-TB cases that they referred to St. Peter Hospital. The same number of controls was selected from each of these five health facilities. The sampling frame comprised all patients who had completed first-line anti-TB treatment and were registered as cured or treatment completed. Following this, the required sample size of the control group was selected using systematic random sampling. When a selected patient declined to participate in the study, the next person in the register was taken.

The contact information of controls and cases was obtained from the health center’s TB clinic patient registration book. The selected individuals were contacted by telephone and given information about the study. Individuals who were willing to participate and gave verbal consent were scheduled for an interview at the health facility.

### Data collection

A structured questionnaire was used to collect information from study participants. Secondary data were collected from TB and MDR-TB registers. Patient charts and the data collection format were used to determine and record their initial TB episode. The FMOH screening tool was used to identify controls free of suspected TB at the time of the study [7]. Day-long training was provided to the nurses and health officers involved in the data collection process. The main variables included in study instrument were sex, age, socioeconomic status, ethnicity, HIV status, adherence or non-adherence to the first course of anti-TB treatment, number of previous anti-TB treatments, treatment with the Category II regimen, ever- interruption in taking medicine for a day, and occurrence of drug side effects during the first course of TB treatment.

### Data management and analysis

Data were entered using Epidata version 3.1 and exported to STATA version 11 for analysis. Data completeness and consistency were checked by running frequencies of each variable. Bivariate analyses were carried out for categorical variables, and odds ratios were used to quantify the strength of association between potential risk factors and MDR-TB. Multiple logistic regressions were used to control the confounding effect of different variables while assessing the effect of each variable on the likelihood of MDR-TB occurrence. A p-value of 0.05 was used as the cut-off point for statistical significance. Variables having a p-value of at most 0.05 in bivariate analysis were included in the multivariate logistic regression model. In multivariate logistic regression, the adjusted effects of three variables (the number of pulmonary TB episodes, ever-interruption in anti-TB treatment for at least a day in the first course, and duration of the first course of TB treatment) were estimated without concurrently adjusting for each other to avoid multicollinearity.

### Ethical considerations

Ethical clearance was obtained from the institutional review board of the Aklilu Lemma Institute of Pathobiology at Addis Ababa University and St. Peter Hospital. Written permission to conduct the study was also obtained from the managers of each health facility. A statement about the purpose of the study was read to each study participant, and those who gave verbal consent to participate in the study were interviewed. Study participants were interviewed privately, and their names were not written on the questionnaire to ensure confidentiality.

## Results

### Sociodemographic characteristics of study participants

A total of 134 cases and an equal number of controls were included in the study. A total of 81 (60.5%) of the MDR-TB cases were males, but females represented the majority in the control group (70 females, or 52.5%). Single or divorced individuals accounted for the majority 101 (75.3%) of the MDR-TB cases but only about half (69, or 51.5%) in the control group (Table 1). The mean age was 25.1 (SD = 10.94) years for MDR-TB cases and 30.72 (SD = 11.4) years for controls.

**Table 1:** Sociodemographic characteristics of MDR-TB cases and their controls in Addis Ababa, 2011

**Characteristics (variables)**	**Cases**		**Controls**	
	**(n = 134)**		**(n = 134)**	
	**Number**	**Percentage**	**Number**	**Percentage**
**Sex**				
Male	81	60.5	64	47.5
Female	53	39.5	70	52.5
**Age at the time of first anti-TB treatment (years)**				
5–25	85	63.4	47	35.1
26–45	40	29.9	70	52.2
46–72	9	6.7	17	12.7
**Marital status**				
Single	85	63.4	60	44.8
Married	32	23.8	56	41.8
Divorced	16	11.9	9	6.7
Widow/widower	1	0.75	9	6.7
**Educational status**				
Up to fourth grade	15	11.2	25	18.6
Completed 5^th^ –8^th^ grade	16	11.9	20	15
Completed 8^th^ –10^th^ grade	27	20.1	25	18.6
Above 10^th^ grade	76	56.7	64	47.8
**Occupation**				
No work	31	23.1	31	23.1
Student	36	26.9	9	6.7
Daily laborer	2	1.5	13	9.7
Government worker	24	17.9	24	17.9
Private worker	31	23.1	42	31.3
Businessman	10	7.5	15	11.2
**Number of rooms in residence**				
1	61	45.5	37	27.6
2–3	57	42.5	63	47
4–5	11	8.2	34	25.4
6–9	5	3.7	0	0
**Family size**				
1–3	57	42.5	49	36.6
4–6	57	42.5	70	52.2
7–11	20	15	15	11.2

### TB-related conditions

Table 2 summarizes TB-related conditions in the cases and controls. Of the 134 MDR-TB cases, 96 (71.6%) had had two or more episodes of TB treatment before they were diagnosed as MDR-TB, and 9 (6.7%) of the cases had had four or more episodes of TB. In the control group, only 14 (10.4%) had undergone two rounds of TB treatment, and one case had suffered four or more episodes of TB. HIV positivity was significantly lower in the MDR-TB cases than in the control group (13.4% versus 29.9%; p-value <0.001). The quality of care provided by health care providers was perceived as poor by 39 (29.1%) of MDR-TB cases and by 3 (2.2%) of the controls.

**Table 2:** Tuberculosis disease-related conditions in each category (case/control) in Addis Ababa, 2011

**Characteristics**	**Cases**		**Controls**	
	**(n = 134)**		**(n = 134)**	
	**Number**	**Percentage**	**Number**	**Percentage**
**No. of pulmonary TB episodes**				
One	29	21.6	119	88.8
Two	66	49.3	14	10.5
Three	30	22.4	0	0
Four or more	9	6.7	1	0.75
**HIV status**				
Negative	116	86.6	94	70.2
Positive	18	13.4	40	29.9
**Ever lived with MDR-TB patient**				
No	122	91.0	134	100
Yes	12	9.0	0	0
**Site of TB infection during first episode**				
Pulmonary	130	97	90	67.2
Extrapulmonary	4	3.0	44	32.8
**Smear-positive during first anti-TB treatment**				
No	11	8.2	82	61.2
Yes	123	91.8	52	38.8
**Ever counseled by health worker**				
No	44	32.8	1	0.75
Yes	90	67.2	133	99.25
**Presence of other disease**				
No	111	82.8	115	85.5
Yes	23	17.2	19	14.2
**Ever smoked cigarettes**				
No	125	93.3	115	85.8
Yes	9	6.7	19	14.2
**Perception about the care provided**				
Very good	5	3.7	87	64.9
Good	13	9.7	36	26.9
Satisfactory	77	55.5	8	6.0
Poor	39	29.1	3	2.2
**Weight measured by health worker before starting treatment**				
No	3	2.2	0	0
Yes	120	89.6	134	100
Doesn’t remember	11	8.2	0	0

### Treatment-related conditions

Conditions related to anti-TB treatment are summarized in Table 3. During first-line anti-TB treatment, drug side effects were encountered in 60 (50%) of the MDR-TB cases and 25 (18.7%) of the controls. Among the current MDR-TB patients, their first-line anti-TB treatment was directly observed by health workers in only 69 cases (51.5%), while 127 (94.8%) of the controls were treated in accordance with the strict DOTS guidelines of the country. First-line anti-TB treatment was interrupted for at least a day in 93 (69.4%) of the MDR-TB cases, and in only 10 (7.5%) of the controls. Out of the 16 MDR-TB patients who were poor adherers of treatment in category I treatment 10(62.5%) were male. Reasons for interruption among MDR-TB cases were drug side effects in 34 cases (36.6%), followed by improved/disappeared symptoms and the perception that TB was cured 29 cases (31.2%), and forgetfulness about taking the medicine 23 cases (24.7%). Duration of first-line anti-TB treatment was exactly 8 months in 103 (76.9%) of the MDR-TB cases and 128 (95.5%) of the controls. Among the MDR-TB cases, the outcomes of the first course of anti-TB treatment was reported as treatment success in 64 cases (47.7%), defaulter in 16 cases (11.9%), and treatment failure in 54 cases (40.3%). In the controls, 132 (98.5%) were declared treatment successes. Of the MDR-TB cases, 107 (79.9%), and 13 (9.7%) of the controls, were treated at least twice with first-line anti-TB treatment. Of the 107 current MDR-TB cases, 101 (94.4%) were also treated with the Category II regimen, while only 3 (23.1%) of the 13 controls were treated with the Category II regimen.

**Table 3:** First-line tuberculosis treatment-related conditions in MDR-TB cases and their controls in Addis Ababa, 2011

**Characteristics**	**Cases**		**Controls**	
	**(n = 134)**		**(n = 134)**	
	**Number**	**Percentage**	**Number**	**Percentage**
**Encountered drug side effect**				
No	67	50.0	109	81.3
Yes	67	50.0	25	18.7
**Suffered the most common drug side effect (vomiting)**				
No	85	63.4	124	92.5
Yes	49	36.6	10	7.5
**Duration of first-time TB treatment**				
2–4 months	3	2.2%	1	0.75%
5–7 months	22	16.4%	2	1.5%
8 months	103	76.9%	128	95.5
9–13 months	6	4.5%	3	2.2%
**Directly observed by health worker while taking anti-TB**				
No	65	48.5	7	5.2
Yes	69	51.5	127	94.8
**If yes, how many months**				
1–2 weeks	11	15.95	0	0
One month	32	46.4	1	0.8
Two months	26	37.7	126	99.2
**Reason for interruption for at least a day**				
Side effects	34	36.6	3	30.0
Forgot to take it	23	24.7	7	70.0
Symptoms were gone and felt good	29	31.2	0	0
Shortage of drug	7	7.5	0	0
**Ever interrupted anti-TB for at least a day**				
No	41	30.6	124	92.5
Yes	93	69.4	10	7.5
**Took the medication at a regular time**				
No	81	60.5	25	18.7
Yes	53	39.5	109	81.3
**Outcome of first anti-TB treatment**				
Treatment success	64	47.7	132	98.5
Defaulted	16	11.9	2	1.5
Treatment failure	54	40.3	0	0
**Drug regimen (category) for the second time**				
Category II	101	94.4	3	23.1
Category I	6	5.6	10	76.9

### Results from logistic regression analysis

After adjusting for possible confounding factors (Table 4), the study found that MDR-TB development is significantly associated with two or more episodes of TB illness (AOR = 31.8; 95% CI; 8.7–115.5), interruption of first-line anti-TB treatment for at least a day (AOR = 13.1; 95% CI; 3.0–56.6), education above 10th grade (AOR = 3.7; 95% CI; 1.1–12.1), and male sex (AOR = 2.7; 95% CI; 1.1–6.5).

**Table 4:** Determinants of multidrug-resistant tuberculosis from logistic regression model

**Characteristics**	**Case**	**Control**	**Crude OR**	**Adjusted OR**
	**Number**	**Number**	**(95% CI)**	**(95% CI)**
**Individually adjusted for the remaining variables**				
**Number of pulmonary TB episodes**				
One	29	119	1	1
Two or more	105	15	28.7 (14.6-56.5)	31.8 (8.7-115.5)
**Ever interrupted anti-TB for at least a day**				
No	41	124	1	1
Yes	93	10	28.1 (13.4-58.1)	13.1 (3.0-56.6)
**Duration of first course of TB treatment (months)**				
2–7	25	3	10.0 (2.9-34.1)	14.8 (2.3-96.4)
≥8	109	131	1	1
**Adjusted for all variables**				
**Age when taking first-line anti-TB for the first time (years)**				
46–72	9	17	1	1
26–45	40	70	1.1 (0.4-2.6)	1.4 (0.3-5.8)
5–25	85	47	3.4 (1.4-8.3)	4.6 (1.1-20.5)
**Marital status**				
Single	85	60	2.5 (1.4-4.3)	1.2 (0.5-3.3)
Married	32	56	1	1
Divorced/separated	17	1	1.7 (0.75-3.7)	3.1 (0.7-13.2)
**Educational status**				
Up to fourth grade	15	24	1	1
Completed 5^th^–8^th^ grade	16	20	1.3 (0.5-3.2)	1.2 (0.3-5.1)
Completed 8^th^–10^th^ grade	27	25	1.7 (0.7- 4.0)	1.4 (0.4-5.1)
Above 10^th^ grade	76	64	1.9 (0.9-3.9)	3.7 (1.1-12.1)
**Sex**				
Female	53	70	1	1
Male	81	64	1.7 (1.0-2.7)	2.7 (1.1-6.5)
**Number of rooms in residence**				
4–9	16	34	1	1
2–3	57	63	1.9 (1.0-3.9)	3.3 (1.0-10.9)
1	61	37	3.5 (1.7-7.2)	10.1 (2.0-49.4)
**Number of rooms in residence**				
4–9	16	34	1	1
2–3	57	63	1.9 (1.0-3.9)	3.3 (1.0-10.9)
1	61	37	3.5 (1.7-7.2)	10.1 (2.0-49.4)
**Family size**				
1–3	57	49	1	1
4–6	57	70	0.7 (0.4-1.2)	1.6 (0.5-5.0)
7–11	20	15	1.2 (0.5-2.5)	2.9 (0.7-13.0)
**HIV status**				
Negative	116	94	2.7 (1.5-5.1)	2.8 (0.9-8.5)
Positive	18	40	1	1
**Site of TB infection during first episode**				
Extrapulmonary	4	44	1	1
Pulmonary	130	90	15.9 (5.5-45.8)	10.9 (2.8-41.9)
**Encountered drug side effect**				
No	67	109	1	1
Yes	67	25	4.4 (2.5-7.6)	4.5 (1.9-10.5)
**Encountered shortage of drug**				
No	106	124	1	1
Yes	28	10	3.3 (1.5-7.1)	2.7 (0.8-9.5)
**Directly observed by health worker while taking anti-TB**				
Yes	69	127	1	1
No	65	7	17.1 (7.4-39.3)	11.7 (4.0-34.3)
**Took the medication at a regular time**				
No	69	127	1	1
Yes	65	7	17.1 (7.4-39.3)	11.7 (4.0-34.3)
**Ever smoked cigarettes**				
No	125	115	1	1
Yes	9	19	0.4 (0.19-1.0)	0.4 (0.1-1.8)

The number of rooms in the patient’s household also showed a significant association with MDR-TB (AOR = 10.1; 95% CI; 2.0–49.4). Pulmonary TB (AOR = 10.9; 95% CI; 2.8–41.9), drug side effects during first-line treatment (AOR = 4.5; 95% CI; 1.9–10.5), lack of direct observation by health workers (AOR = 11.7; 95% CI; 4.0–34.3), and less than 7 months of first-line anti-TB treatment (AOR = 14.8 95% CI; 2.3–96.4) were also significantly associated with MDR-TB development. Fischer’s exact test showed that being treated with the Category II regimen was associated with MDR-TB development (P = 0.000).

HIV status, history of smoking, experience of drug shortages, and family size were not significantly associated with MDR-TB development.

## Discussion

A case control study with equal number of cases and controls was conducted by recruiting a total of 268 study participants to determine factors associated with developing MDR-TB after taking first line anti-TB treatment. Factors which were associated with MDR-TB: the first site of TB infection being pulmonary, encountering drug side effects during the first course of treatment, having more than one TB episode, undergoing the Category II regimen, and taking anti-TB treatment for less than 7 months.

The study also found that being male was a risk factor for MDR-TB development. A study in Nigeria showed that being male was a risk factor for defaulting from anti-TB medication [14]. Similarly, this study showed that among MDR-TB cases who were defaulters in their first-line TB treatment, 62.5% were males. The association between being male and having MDR-TB could be due to the fact that males have a higher tendency not to adhere to anti-TB treatment than females, thus increasing their risk of developing MDR-TB. Another study showed that individuals who do not take anti-TB medication regularly have increased risk for MDR-TB [15]. Our study also showed that individuals who did not take first-line anti-TB drugs regularly had increased risk for development of MDR-TB.

Evidence from a previous study has shown that poor treatment adherence was a risk factor for MDR-TB [8]. The current study also showed that individuals who took first-line anti-TB treatment for duration of 2 to 7 months had increased risk of developing MDR-TB. In Ethiopia, the previous guideline for first-line anti-TB treatment was 8 months’ duration, but the standard has been changed to 6 months. TB therapy requires more than 90% adherence to facilitate cure [16], and 2 to 7 months (25%–87.5% of the prescribed duration) is less than the required duration to result in cure.

Additionally, individuals who were not under strict DOTS per national guidelines during their first anti-TB treatment had an 11.7 times increased risk for MDR-TB. An analysis that used empirical data to determine the impact of the expansion of the DOTS strategy on TB case finding and treatment success found that countries with full DOTS coverage had at least an 18% increase in the treatment success rate [17]. An individual who is supervised by a health worker is more likely to take the appropriate dose of medicine and less likely to miss a treatment. Furthermore, individuals who come for DOTS have frequent contact with health workers and thus have increased opportunities to get advice and counseling, which might help them to adhere to medication protocol.

As expected, individuals who encountered drug side effects during the first course of TB treatment had a 4.5 times increased risk of developing MDR-TB. Studies done in three districts of Arsi Zone, Ethiopia, found that anti-TB drug side effects were significantly associated with a high rate of defaulting [18]. When patients develop side effects, they tend to stop treatment, which favors the development of MDR-TB. If the DOTS strategy of the nation were followed in all cases, there would be a chance to counsel patients and even treat adverse drug reactions before treatment interruption. In our study, the first-line anti-TB treatment of 48.5% of the MDR-TB cases was not directly observed. A systematic review of 29 published reports on risk factors associated with MDR-TB in Europe revealed that previous treatment was the strongest determinant of MDR-TB and that the pooled risk of MDR-TB was 10.23 times higher in previously treated than in never-treated cases [19]. A study in Uganda also showed that multiple TB episodes and treatment failure were significantly associated with MDR-TB [20]. Similarly, in Ethiopia, according to a nationwide anti-TB drug resistance survey conducted in 2005, 1.6% of newly diagnosed TB cases were infected with MDR-TB, while 11.8% of the MDR-TB cases were previously treated TB cases [10].

One can see how MDR-TB is prevalent in individuals who have a history of treatment compared to new patients. Similarly, the current study showed that having more than one TB episode also increased risk for MDR-TB. This may be related to the previous treatment outcome, default, treatment failure, or relapse, or the patient may have had MDR-TB initially.

Having pulmonary TB during first anti-TB treatment was associated with increased risk for MDR-TB. This may also be associated with the fact that smear-positive pulmonary TB individuals have a high bacterial load and may not respond to the treatment within a short period of time, as do those with a low bacterial load [21]. For this reason, smear-positive pulmonary TB patients might be more prone to develop MDR-TB. The other explanation might be associated with diagnostic difficulties. In case of extra pulmonary MDR- TB the bacterial load is lower and difficult for definite diagnosis comparing to pulmonary MDR-TB. Limited capacity of the existing laboratory facilities especially for the diagnosis of extra pulmonary MDR-TB might explain the association of being Pulmonary TB and having MDR-TB.

This study showed that individuals who were treated by the Category II regimen had increased risk for MDR-TB. More than one explanation may be given for the association of Category II treatment and MDR-TB. These individuals might have had a previous TB treatment history and registered for the treatment as treatment failures, defaulters, or relapse cases, or they might have already had MDR-TB at the initiation of the Category II regimen. Another explanation is that adding one drug in the failing regimen could change susceptible strains and lead to multidrug resistance. “Michael Iseman, the US-based MDR-TB specialist, had 10 commandments for the physicians not to change fully drug susceptible organisms to MDR-TB; the first one was never to add a single drug to a failing regimen and the other nine were to repeat the first commandment to make sure it was well understood” [8]. WHO recommends that DST should be done for all previously treated patients before they are treated with the Category II drug regimen, and in conditions where DST is not available, the Category II regimen can be used for relapse, default, and treatment failure for low- or medium-MDR-TB-burden countries [9]. A cross-sectional study in South Africa showed that retreatment patients had increased risk for any drug resistance and MDR-TB [22]. Having a DST before embarking on the Category II regimen is very important. In Ethiopia, because of low laboratory capacity, performing DST for all previously treated patients is difficult even though the country is one of the high-MDR-TB- burden countries. An individual’s treatment may fail because they have already had MDR-TB or because drug resistance was caused by the retreatment regimen [23]. This is because the patient has already taken all the drugs in the Category II regimen in the previous treatment, except streptomycin, which is the oldest drug.

In the current study, HIV status had no significant association with MDR-TB. A study in Thailand showed also that HIV status was not significantly associated with MDR-TB [23]. In France, being HIV positive was associated with primary MDR-TB but it was not associated with secondary MDR-TB [24]. A cross-sectional study in South Africa showed that in retreated patients, HIV had no significant association with MDR-TB [25]. The study participants in the current study were patients who had a history of first-line anti-TB treatment. It is possible that the result could have been different if all study participants were primary MDR-TB cases rather than MDR-TB cases who had a history of previous treatment. A study in Ukraine showed that HIV-positive individuals had a 50% higher risk of developing MDR-TB at their first TB infection [26]. This is because being HIV positive is one risk factor for drug-susceptible TB, which is related to immune system suppression. Being HIV positive might carry the same risk of infection with MDR-TB but may not contribute to the change of a drug-susceptible strain of TB to MDR-TB.

The strengths of the current study are that study participants in the control group finished first-line anti-TB treatment two years before the study period, which reduced the chance of relapse. They were selected from the five health facilities in Addis Ababa that reported the most MDR-TB cases to St. Peter Hospital, so that cases and controls would have a better likelihood of coming from similar backgrounds and be most likely to receive the same service. Regarding the case group, all cases that fulfilled the eligibility criteria that were available during the study period and willing to respond were included in the study. This was helpful to decrease sampling error.

The current study is not without limitations, however. Recall bias could be considered one potential challenge, since some of the information was based on the recall of the study participants. Furthermore, it was not clear whether all cases had MDR-TB before or after undergoing first-line TB treatment, since DST was not done before they took first-line TB treatment or Category II regimens.

## Conclusions

Non-adherence to the first line anti-TB treatment was significantly associated with MDR-TB. Taking medication without interruption, taking medication regularly, and having supervision (DOTS) had a protective effect against MDR-TB. Having more than one pulmonary TB episode had a significant association with MDR-TB. Individuals who were treated with the Category II regimen were also found to have an increased risk for MDR-TB. HIV status was not significantly associated with MDR-TB among individuals who had been previously treated with first-line anti-TB drugs. Hence, strengthening DOTS programs to enhance patient adherence to anti-TB treatment and giving special attention to individuals at high risk for MDR-TB and prioritizing them for DST are recommended.

## Abbreviations

AFB: Acid fast bacilli; AOR: Adjusted odds ratio; CI: Confidence interval; DOTS: Directly observed treatment short course; DST: Drug sensitivity test; EHNRI: Ethiopia health nutrition and research institute; FMOH: Federal Ministry of Health; HBC: High burden country; HIV: Human immune deficiency virus; IRB: Institutional Ethical Review Board; MDR-TB: Multi drug resistant tuberculosis; OR: Odds ratio; PI: Principal investigator; TB: Tuberculosis; WHO: World Health Organization; XDR-TB: Extensive drug resistant tuberculosis.

## Competing interests

All authors declare that they have no competing interests.

## Authors’ contributions

SH conceived the idea of the study, prepared the study proposal, collected data in the field, performed the data analysis, and drafted the manuscript. GA and GM assisted with the preparation of the proposal and the interpretation of data, participated in data analysis, and critically reviewed the manuscript. BG participated in the proposal preparation, interpretation of data, and critical review of the manuscript. AM participated in the interpretation of data and critically reviewed the manuscript. MM and PS critically reviewed the proposal and the manuscript. All authors read and approved the final manuscript. All authors participated in critical appraisal and revision of the manuscript.

## Pre-publication history

The pre-publication history for this paper can be accessed here:

http://www.biomedcentral.com/1471-2458/13/782/prepub
